# Epicatechin gallate, a naturally occurring polyphenol, alters the course of infection with β-lactam-resistant *Staphylococcus aureus* in the zebrafish embryo

**DOI:** 10.3389/fmicb.2015.01043

**Published:** 2015-09-28

**Authors:** Christina S. Stevens, Helena Rosado, Robert J. Harvey, Peter W. Taylor

**Affiliations:** UCL School of Pharmacy, University College LondonLondon, UK

**Keywords:** epicatechin gallate, tea catechins, MRSA, *Staphylococcus aureus*, zebrafish embryo infection, antibacterial activity, respiratory burst

## Abstract

(-)-epicatechin gallate (ECg) substantially modifies the properties of *Staphylococcus aureus* and reversibly abrogates β-lactam resistance in methicillin/oxacillin resistant (MRSA) isolates. We have determined the capacity of ECg to alter the course of infection in zebrafish embryos challenged with epidemic clinical isolate EMRSA-16. At 30 h post fertilization (hpf), embryos were infected by injection of 1–5 × 10^3^ colony forming units (CFU) of EMRSA-16 into the circulation valley or yolk sac. Infection by yolk sac injection was lethal with a challenge dose above 3 × 10^3^ CFU, with no survivors at 70 hpf. In contrast, survival at 70 hpf after injection into the circulation was 83 and 44% following challenge with 3 × 10^3^ and 1–5 × 10^3^ CFU, respectively. No significant increases in survival were noted when infected embryos were maintained in medium containing 12.5–100 μg/mL ECg with or without 4 or 16 μg/mL oxacillin. However, when EMRSA-16 was grown in medium containing 12.5 μg/mL ECg and the bacteria used to infect embryos by either the circulation valley or yolk sac, there were significant increases in embryo survival in both the presence and absence of oxacillin. ECg-modified and unmodified, GFP-transformed EMRSA-16 bacteria were visualized within phagocytic cells in the circulation and yolk sac; pre-treatment with ECg also significantly increased induction of the respiratory burst and suppressed increases in IL-1β expression typical of infection with untreated EMRSA-16. We conclude that exposure to ECg prior to infection reduces the lethality of EMRSA-16, renders cells more susceptible to elimination by immune processes and compromises their capacity to establish an inflammatory response in comparison to non-exposed bacteria.

## Introduction

*Staphylococcus aureus* is a highly successful opportunistic pathogen: it is a common component of the microbiota of the upper respiratory tract and skin (Foster, 2004) but may also cause a variety of nosocomial and community-acquired infections, ranging from minor skin conditions to life-threatening diseases such as endocarditis, septicemia and toxic shock syndrome (Plata et al., 2009; Thwaites et al., 2011). *S. aureus* also has the capacity to accumulate antibiotic resistance genes. Infections due to multi-drug-resistant forms such as methicillin-resistant *S. aureus* (MRSA) can occur in epidemic waves that are initiated by one or a few successful clones and can spread rapidly among hospitalized patients and healthy individuals in the community alike (Chambers and DeLeo, 2009). MRSA isolates are invariably resistant to all β-lactam agents due to the acquisition of *mecA* or its homolog *mecC*. These genes encode the low-affinity penicillin binding protein (PBP) 2a, a transpeptidase that forms a functional complex with PBP2 to enable peptidoglycan synthesis after β-lactam acylation of native, membrane-localized PBPs (Fuda et al., 2005; Paterson et al., 2014). MRSA infections are associated with extended hospital stay, high treatment costs, prolonged illness and adverse mortality rates (Datta and Huang, 2008; Anderson et al., 2009) and new treatments and prophylactic measures are urgently needed. Novel treatment options that reduce the rate of emergence of drug resistance would be particularly welcome.

The naturally occurring, abundant polyphenol (-)-epicatechin gallate (ECg) has the capacity to abrogate β-lactam resistance in MRSA, reduce the secretion of virulence effectors such as toxins and tissue-degrading enzymes, and prevent the formation of biofilms (Taylor et al., 2005; Taylor, 2013), making it potentially useful for the control of difficult-to-treat staphylococcal infections. In common with catechins and other catechin gallates, ECg shows a strong tendency to partition into lipid bilayers, including model lipid bilayers comprising single phospholipid species and more complex biological membranes such as the staphylococcal cytoplasmic membrane (CM) (Caturla et al., 2003; Bernal et al., 2010). ECg penetrates deep into the hydrophobic core of the lipid palisade of the staphylococcal bilayer to induce a comprehensive reconfiguration of membrane architecture (Palacios et al., 2014; Rosado et al., 2015), providing a suboptimal environment for the cell wall biosynthetic and cell division machineries. Thus, intercalation of ECg into the staphylococcal CM leads to increased cell wall thickness (Stapleton et al., 2007) and increases in the net negative charge at the bacterial surface due to reductions in D-alanylation of wall teichoic acid (Bernal et al., 2009) and lysylation of phosphatidylglycerol head groups (Bernal et al., 2010; Rosado et al., 2015). Most importantly, ECg intercalation delocalizes PBP2 from the septal site of cell division (Bernal et al., 2010) and disrupts the functional integrity of the cell division assemblage, the divisome (Paulin et al., 2014). These latter effects account for the reversible loss of β-lactam resistance induced by ECg.

The oral bioavailability of naturally occurring catechin gallates in humans and other animals is low (Yashin et al., 2012), they are poorly absorbed from the intestinal tract and are rapidly metabolized to inactive products due to the presence of ester bonds susceptible to enzymatic hydrolysis (Kohri et al., 2001). Further, they appear in the blood circulation mainly in conjugate form after glucuronidation, sulfation, or methylation (Yang et al., 2008). They also display chemical instability (Zhu et al., 1997). Degradation of ECg in biological milieu can be prevented by replacement of a hydrolytically susceptible ester linkage with a stable amide whilst maintaining the natural stereochemistry (Anderson et al., 2005a) and membrane penetration can be enhanced by removal of hydroxyl functions on one of the two ECg pharmacophores (Anderson et al., 2005b, 2011). However, even after optimization of the routes to catechin stereochemistry (Anderson et al., 2014), chemical synthesis of these catechin analogs is sufficiently complex and low-yielding for the generation of adequate quantities of material to enable profiling in small animal models of infection to be unrealistic. We have therefore adapted a model of staphylococcal infection in the zebrafish embryo (Prajsnar et al., 2008) to gain insights into the capacity of ECg to alter the *in vivo* course of infection with MRSA.

Zebrafish are small tropical freshwater fish native to India, Pakistan, and Bhutan and have provided a powerful model for the study of developmental biology and disease (Goldsmith and Jobin, 2012). External fertilization, *ex utero* development and the transparency of zebrafish embryos enables the details of embryological processes and development to be investigated using a light microscope, in contrast to the mouse, in which this stage occurs *in utero* (Stuart et al., 1990). The transparency of zebrafish embryos also allows for fluorescent dyes to be observed in live embryos by microscopy (Herbomel et al., 1999). Embryos possess functional innate immunity and have facilitated the dissection of non-specific host-pathogen interactions during staphylococcal infection (Prajsnar et al., 2008). Here we show that pre-treatment with ECg reduces the lethality of MRSA for zebrafish embryos in both the presence and absence of the β-lactam oxacillin.

## Materials and Methods

### Bacteria

Epidemic MRSA strain EMRSA-16 was isolated from a clinical sample obtained at the Royal Free Hospital, London. EMRSA-16 expressing Green Fluorescent Protein (EMRSA16-GFP) was obtained by transformation of electro-competent *S. aureus* EMRSA-16 cells with plasmid pSB2035 (P3 amplified by PCR from *S. aureus* 8325-4, exchanged with P_xylA_ from pSB2030, Ap^r^ Cm^r^; Qazi et al., 2001) DNA extracted from *S. aureus* SJF1219, kindly provided by Professor Simon Foster (Sheffield University, UK). Bacteria were grown in Mueller Hinton (MH) broth at 37°C to mid-logarithmic phase (OD_600_ 0.7) with agitation in an orbital incubator (200 orbits/min) and collected by centrifugation. Bacterial pellets were suspended in phosphate buffered saline (PBS) containing 0.05% sterile phenol red (Sigma–Aldrich, Gillingham, Dorset, UK), or PBS alone for fluorescence assays. Both were filtered through a 0.22 μm Millex filter (Millipore, Carrigtwohill, Ireland). Bacteria were enumerated by serial dilution and plating on to MH agar or MH agar containing 20 μg/mL chloramphenicol (for EMRSA16-GFP). Growth medium was supplemented as required with ECg, provided by Mitsui Norin Co., Tokyo, Japan. ECg was dissolved in 50% v/v ethanol and added to the bacterial culture to a final concentration of 12.5 μg/mL as required.

### Zebrafish Husbandry

Wildtype AB/TULF zebrafish were maintained at the University College London zebrafish facility (http://www.ucl.ac.uk/zebrafish-group) in a multi-rack recirculating system from Aquatic Habitat (Apopka, FL, USA) at an air temperature of 24°C, water temperature of 28.5°C and pH of 7.6. Adult zebrafish were maintained in 10 L tanks, containing approximately 9 L of filtered, recirculated tap water, with a maximum density of 30 fish. Fish were fed three times daily with a mixture of brine shrimp, krill, and hikari high protein pellets and were daily monitored for signs of disease. Zebrafish were maintained on a 14 h light and 10 h dark photoperiod and embryos collected within 1 h of the onset of the light cycle. Embryos were incubated at 28.5°C in E3 medium according to Nüsslein-Volhard and Dahm (2002).

### Microinjection of Zebrafish Embryos

TW100-4 borosilicate glass capillaries [World Precision Instruments (WPI), Sarasota, FL, USA] were pulled in a Model P-97 Flaming/Brown Micropipette Puller (Sutter Instruments Inc, Novato, CA, USA). The injection volume was determined by expansion upon injection of bacterial suspension into oil on a graticule. Embryos were mechanically dechorionated 24 h post fertilization (hpf) using jewelers’ forceps and anesthetized by submersion in 0.02% (w/v) buffered (pH 7.0) tricaine. Embryos were embedded in 3% (w/v) methylcellulose and injected individually with 1–2 nL of bacterial suspension into the circulation valley or the yolk sac using a rig consisting of a pneumatic micropump, microinjector, micromanipulator, injection arm (all WPI), and optical dissecting microscope. Infected embryos were carefully washed and each placed in one well of a 96-well microtiter dish and incubated at 28.5°C. The bacterial suspension was prepared with phenol red in order to visualize and confirm the site of injection and volumes injected into PBS and viable counts performed to determine the precise injected inoculum. In some experiments, embryos were incubated with ECg and oxacillin. The concentrations of these compounds in the E3 incubation mixture were determined by HPLC (1260 Infinity system, Agilent Technologies, Wokingham, UK) using a Synergi Polar RP 250 mm × 4.6 mm, 4 mm column (Phenomenex, Macclesfield, UK). The mobile phase, optimized for clear separation of components of interest, contained 25% acetonitrile in 0.1% trifluoroacetic acid; an injection volume of 10 μL and a flow rate of 1 mL/min with UV detection (280 nm) were employed. Embryos up to 120 hpf obtain nutrients exclusively from the yolk sac and do not fall under U.K. or European regulatory frameworks dealing with animal experimentation.

### Embryo Mortality and Bacterial Enumeration Following EMRSA-16 Infection

EMRSA-16 [1–5 × 10^3^ colony forming units (CFU)] was injected into the circulation valley or yolk sac of groups of ∼30 zebrafish embryos at 30 hpf. Control embryos were either injected with 0.05% phenol red in PBS or heat-killed (80°C for 30 min) EMRSA-16. Embryos were placed individually into wells of a 96-well microtiter dish after injection of bacteria, incubated at 28.5°C and inspected for survival at regular intervals up to 70 h post infection (hpi). Survival was determined by microscopic observation of the heartbeat; deaths were recorded at each time point. At regular intervals up to 70 hpi, live and dead embryos were placed individually into 100 μL of E3 medium, homogenized with a micropestle, serially diluted and plated on to MH agar to enumerate EMRSA-16. As embryos had developed a microbiota by 30 hpf (unpublished; and see Roeselers et al., 2011), 50 μg/mL kanamycin was incorporated into the growth medium; EMRSA-16 is kanamycin-resistant.

### Microscopy

EMRSA-16-GFP was visualized within anesthetized (0.02% tricane) embryos using a Zeiss LSM 710 confocal microscope (Carl Zeiss, Oberkochen, Germany) fitted with a 25mW 488 nm Argon ion laser.

### Determination of Respiratory Burst

Respiratory burst was determined essentially according to Hermann et al. (2004). Individuals from groups of ten embryos were placed in wells of a 96-well microtiter dish with 100 μL E3 medium and the production of reative oxygen species (ROS) stimulated with 400 ng/mL phorbol mysristate acetate (PMA; in DMSO) in the presence of 1 μg/mL of the non-fluorescent dye 2′,7′-dihydrodichlorofluorescein diacetate (H2DCFDA; Invitrogen, Paisley, UK). Oxidation of H2DCFDA produces the fluorescent product dichlorofluorescein, which was detected by excitation at 485 nm and emission at 520 nm. Wells containing embryos in suspension were normalized to wells containing only suspension medium and data normalized to background fluorescence.

### Analysis of Fluorescent Cell Populations by Flow Cytometry

Embryo cell blood cell populations were examined by flow cytometry. Groups of one hundred embryos were anesthetized, collected and as much E3 medium as possible removed. Isotonic calcium-free Ringer’s solution (100 μL) was added, yolks were removed by mixing with a P200 pipette and the suspension incubated for 5 min at room temperature; 2 mL of 0.05% trypsin was then added, the mixture incubated for 30 min at 30°C and embryos dissociated by pipetting at regular intervals (every 5–10 min) with a P1000 pipette. This lysis step was terminated by addition of 200 μL of 2 mM CaCl_2_ and 100 μL of 1% w/v bovine serum albumin. The cell suspension was filtered through a 40 μm cell strainer to ensure that it would not block the flow cytometer and centrifuged at 300 *g* for 7 min at 20°C. The supernatant was removed and centrifuged at 5000 *g* for 10 min. Pellets from both centrifugation steps were suspended in 1 mL of PBS, combined and incubated with 10 μg/mL of propidium iodide (PI) in the dark for 15 min (PI is membrane impermeant, excluded from viable cells, and identifies dead cells; absorption maximum 535 nm, emission maximum 617 nm). Cells were analyzed using MACSQuant^®^ Analyzer (Milteni Biotech, Bergisch Gladbach, Germany); 20 000 events were detected in an uptake volume of 200 μL. Forward scatter was set to 300 V and side scatter to 330 V. All experiments were undertaken ten times.

### Quantitative Real-time PCR (qRT-PCR)

Transcription of genes encoding β-actin, IL-1β, and TNF-α was determined by qRT-PCR, essentially as described previously (Birchenough et al., 2013) using the following forward and reverse primers: β-actin, ATG GAT GAG GAA ATC GCT G & ATG CCA ACC ATC ACT CCC TG; IL-1β, CGC CCT GAA CAG AAT GAA GCA C & AAG ACG GCA CTG AAT CCA CCA C; TNF-α, GGG CAA TCA ACA AGA TGG AAG & GCA GCT GAT GTG CAA AGA CAC.

## Results

### EMRSA-16 Infection of Zebrafish Embryos

**FIGURE 1:**
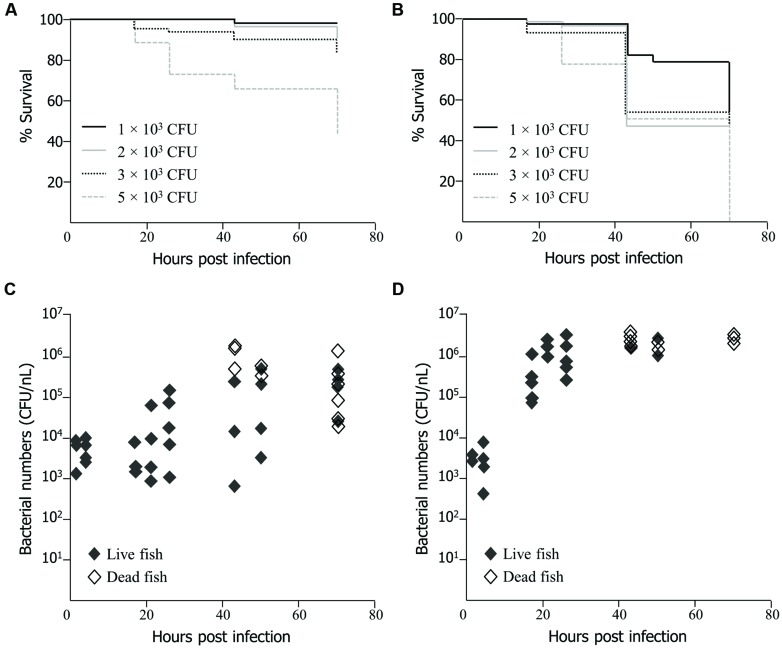
**Survival of zebrafish embryos following injection at 30 h post fertilization (hpf) with EMRSA-16 bacteria into the circulation valley **(A)** or yolk sac (B).** *n* = 21 ± 1 in each group for **(A)**, *n* = 22 ± 2 for **(B)**. The number of bacteria injected in a volume of 1–2 nl ranged from 1 × 10^3^ to 5 × 10^3^ colony forming unit (CFU), as indicated. The staphylococcal bioburden in embryos infected by the circulation valley at 30 hpf with 5 × 10^3^ CFU is shown in **(C)** and by the yolk sac with 2 × 10^3^ CFU in **(D)**. *n* = 60 for each group.

EMRSA-16 cells were injected into the yolk sac and circulation valley at 30 hpf, as established by Prajsnar et al. (2008). Survival of embryos infected by the circulation valley was directly related to the number of injected bacteria (**Figure 1A**) as determined by product limit estimation (Kaplan and Meier, 1958). Death occurred earlier in embryos infected with 3 × 10^3^ and 5 × 10^3^ CFU of EMRSA-16 than in embryos infected with 1 × 10^3^ and 2 × 10^3^ CFU. At 70 hpi 95% of embryos infected with1 × 10^3^ CFU survived, compared with 87% infected with 2 × 10^3^, 83% with 3 × 10^3^ and 44% with 5 × 10^3^ CFU. All embryos injected with the same volume of 0.05% phenol red in PBS or heat-killed EMRSA-16 survived (data not shown). A similar trend was evident when EMRSA-16 was injected into the yolk, with survival decreasing in a dose-dependent manner (**Figure 1B**). Deaths were recorded in each infected group at 17 hpi and a subsequent decrease in survival at each time point was observed. A substantial decrease in survival was detected at 43 hpi in embryos infected with 2 × 10^3^, 3 × 10^3^, and 5 × 10^3^ CFU. After 70 hpi, 54% of embryos infected with 1 × 10^3^ and 50% with 2 × 10^3^ CFU survived. However, no embryos survived in the groups infected with 3 × 10^3^ or 5 × 10^3^ CFU.

To determine the extent of EMRSA-16 proliferation following infection, homogenates of live and dead zebrafish embryos were enumerated by serial dilution and subsequent cultivation on MH agar containing kanamycin. The size of the infecting dose selected for these experiments reflected the differences in the dose-dependent lethal effect between yolk-infected and circulation-infected embryos. With both routes of infection, the bacterial bioburden tended to increase over time. Injection of 5 × 10^3^ CFU into the circulation valley indicated that in some, but not all, embryos there was a 1-2-log increase in viable bacteria over a 70 hpi period (**Figure 1C**). In some embryos, a decrease in bacterial load was evident, in all probability reflecting the capacity of a proportion of infected embryos to recover. There was clear evidence of bacterial proliferation in all embryos that died as a result of infection. The bacterial load was particularly widely distributed at 43 hpi, with mean counts from live embryos ranging from 5.75 × 10^2^ to 2.15 × 10^5^ CFU. Deaths began to occur at 43 hpi and were evident at each subsequent time point. A mean of 9.8 × 10^5^ CFU was detected in dead embryos at 70 hpi. A large number of dead fish were identified at 70 hpi, although some embryos were alive at this time point. The lethal effect was more evident when EMRSA-16 was injected into the yolk sac and temporal aspects of bacterial load were therefore examined following administration of 2 × 10^3^ CFU (**Figure 1D**). The bioburden increased rapidly over the first 30 hpi and deaths began to appear at 43 hpi. The mean number cultured from dead embryos was 2.6 × 10^6^ CFU at 43 hpi compared to a mean from live embryos of 2.2 × 10^6^ CFU. By 70 hpi, a large number of fish had died, with a mean EMRSA-16 burden of 2.8 × 10^6^ CFU. In this representative experiment only three live fish remained at this time point.

### Impact of ECg and Oxacillin on Zebrafish Embryo Survival

Growth in liquid medium containing ECg has a significant, reversible impact on the minimum inhibitory concentration (MIC) of β-lactam antibiotics against EMRSA-16 and other resistant strains of *S. aureus* (Stapleton et al., 2004). For example, after 4 h growth in 12.5 μg/mL of ECg, the MIC of EMRSA-16 for oxacillin is reduced from 512 to 1 μg/mL (Rosado et al., 2015). We therefore investigated the impact of exposure to ECg alone and ECg in combination with oxacillin on the survival of 30 hpf embryos; the concentrations of oxacillin employed reflected those that were efficacious following conversion of EMRSA-16 to phenotypic susceptibility by ECg *in vitro*. No deaths or deformities were observed when embryos were exposed to ECg over a range of 12.5–100 μg/mL or to oxacillin over 4–300 μg/mL (data not shown). EMRSA-16 cells were injected into the circulation valley (5 × 10^3^ CFU) or yolk sac (3 × 10^3^ CFU) of each member of groups of 20 embryos and each embryo placed in individual wells containing 12.5, 50, or 100 μg/mL of ECg without oxacillin or with 4 or 16 μg/mL of the antibiotic. No significant increases in survival were found in the presence of ECg alone or in combination with oxacillin at any concentration (all *P* > 0.05; log rank test) regardless of the site of injection (data not shown). Incubation of 30 hpf embryos infected by the circulation valley or yolk sac with chloramphenicol (10 μg/mL), erythromycin (10 μg/mL), kanamycin (100 μg/mL), or vancomycin (200 μg/mL) in the absence of ECg failed to increase embryo survival.

**FIGURE 2:**
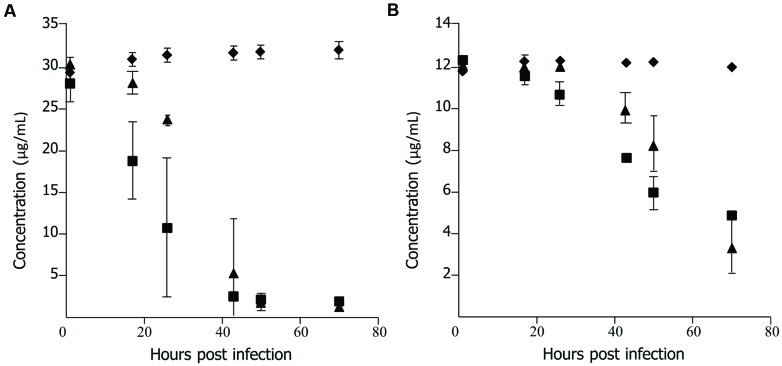
**Uptake of epicatechin gallate (ECg) and oxacillin by uninfected 30 hpf zebrafish embryos incubated at 28.5°C in E3 medium. (A)** Uptake of ECg: 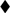, control, E3 medium containing 37.5 μg/mL ECg and 12 μg/mL oxacillin, no embryos; 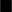, untreated embryos in E3 medium containing 37.5 μg/mL ECg and 12 μg/mL oxacillin; 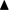, sham treated (PBS) embryos in E3 medium containing 37.5 μg/mL ECg and 12 μg/mL oxacillin. (*n* = 150 per group) **(B)**, uptake of oxacillin: symbols as for **(A)**; *n* = 150 per group. Each concentration determined by HPLC on five occasions; mean ± SD.

To determine if lack of efficacy of ECg was due to a failure of uptake of the polyphenol by the fish, uninfected embryos (untreated and PBS sham-injected) were incubated in E3 medium containing 37.5 μg/mL ECg and 12 μg/mL oxacillin, the samples removed and the concentrations of ECg and oxacillin in the medium quantified by HPLC. No components of E3 medium masked peaks attributable to the test substances (data not shown). Each group of 150 embryos was incubated in a single petri dish. Representative data is shown in **Figure 2**. There were large, significant reductions in the concentrations of both ECg and oxacillin in the presence of pooled embryos. A significant decrease in ECg concentration was found after 17 h incubation with untreated embryos and a smaller but significant effect at this time point was evident with sham-injecteded embryos (**Figure 2A**). At 70 h, less than 5% of the initial concentration of ECg was detected in E3 medium. There were significant reductions in the oxacillin concentration in solution after 17–26 h incubation and the concentrations continued to decline over the 70 h incubation period. Up to 50 hpi, the oxacillin concentration declined more rapidly with untreated embryos in comparison to sham-injected fish (**Figure 2B**).

**FIGURE 3:**
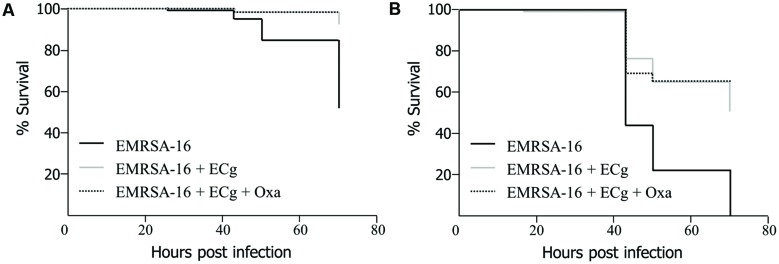
**Survival of zebrafish embryos following injection into the circulation valley **(A)** or yolk sac **(B)** at 30 hpf with 3 × 10^3^ EMRSA-16 bacteria modified *in vitro* by growth in 12.5 μg/mL ECg and incubation in antibiotic-free E3 medium or E3 medium containing 4 μg/mL oxacillin**. *n* = 23–24 per group; differences between ECg-modified and unmodified bacteria were significant for both injection sites *P* < 0.001 (log rank test).

To obtain evidence that MRSA cells modified by exposure to ECg are impaired with regard to their capacity to cause lethal infection, we grew EMRSA-16 to mid-logarithmic phase in MH broth supplemented with 12.5 μg/mL ECg and infected 30 hpf embryos by the circulation valley (**Figure 3A**) or yolk sac (**Figure 3B**) with 3 × 10^3^ CFU of ECg-modified bacteria; infected embryos were then incubated in E3 medium with and without oxacillin (4 μg/mL) supplementation. For both injection sites, ECg-modified bacteria were significantly less virulent than EMRSA-16 cells that were not exposed to ECg prior to administration. Incubation with oxacillin after infection had no impact of survival (circulation: *P* = 0.5627; yolk: *P* = 0.6129; log rank test).

### Impact of EMRSA-16 Infection on Immune Parameters of Zebrafish Embryos

**FIGURE 4:**
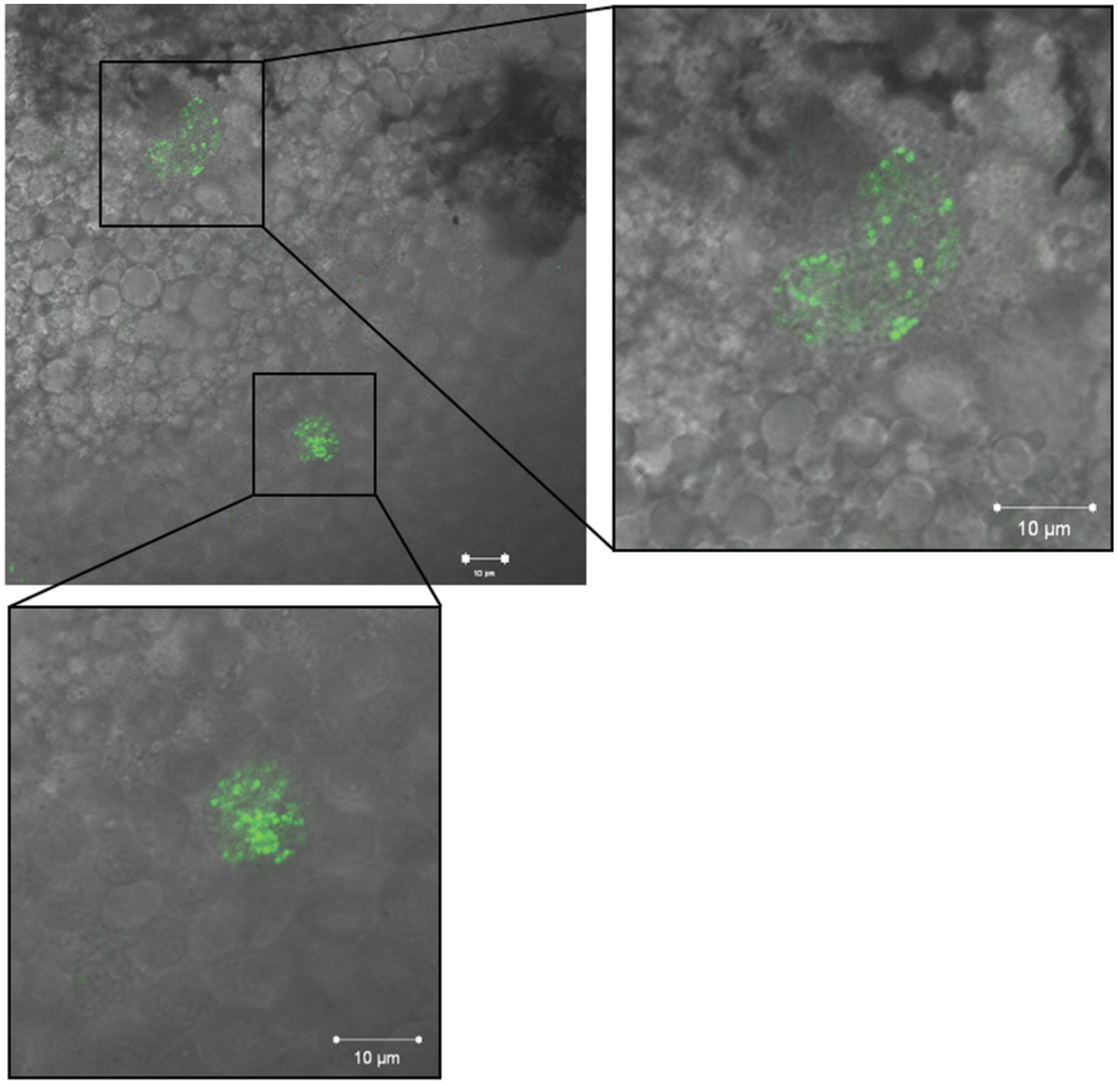
**Fluorescence imaging of EMRSA-16-GFP in cells with granulocyte appearance of the yolk sac of zebrafish embryos infected by the circulation valley at 30 hpf; image obtained 26 h post infection (hpi)**. Scale bar 10 μm.

We employed EMRSA-16-GFP in order to visualize bacteria within 30 hpf embryos following injection into the circulation. Introduction of the GFP cassette into EMRSA-16 elicited a small but significant increase in embryo survival (data not shown); therefore, for these experiments an inoculum of 1 × 10^4^ CFU was employed. At 26 hpi, fluorescent bacteria were visible in the circulation valley and lining blood vessels. The large majority of bacteria were found within cells resembling phagocytes. **Figure 4** shows typical clusters within granulocyte-like cells of the yolk sac; large numbers of bacteria were found in this anatomical site after administration into the circulation. Growth of EMRSA-16-GFP in ECg-containing E3 medium attenuated the bacteria in similar fashion to that found with the non-fluorescent parent strain but no differences were noted in the distribution of ECg-treated and untreated fluorescent bacteria over the 70 h observation period. At 70 hpi, only very few fluorescent bacteria could be detected in survivors.

**FIGURE 5:**
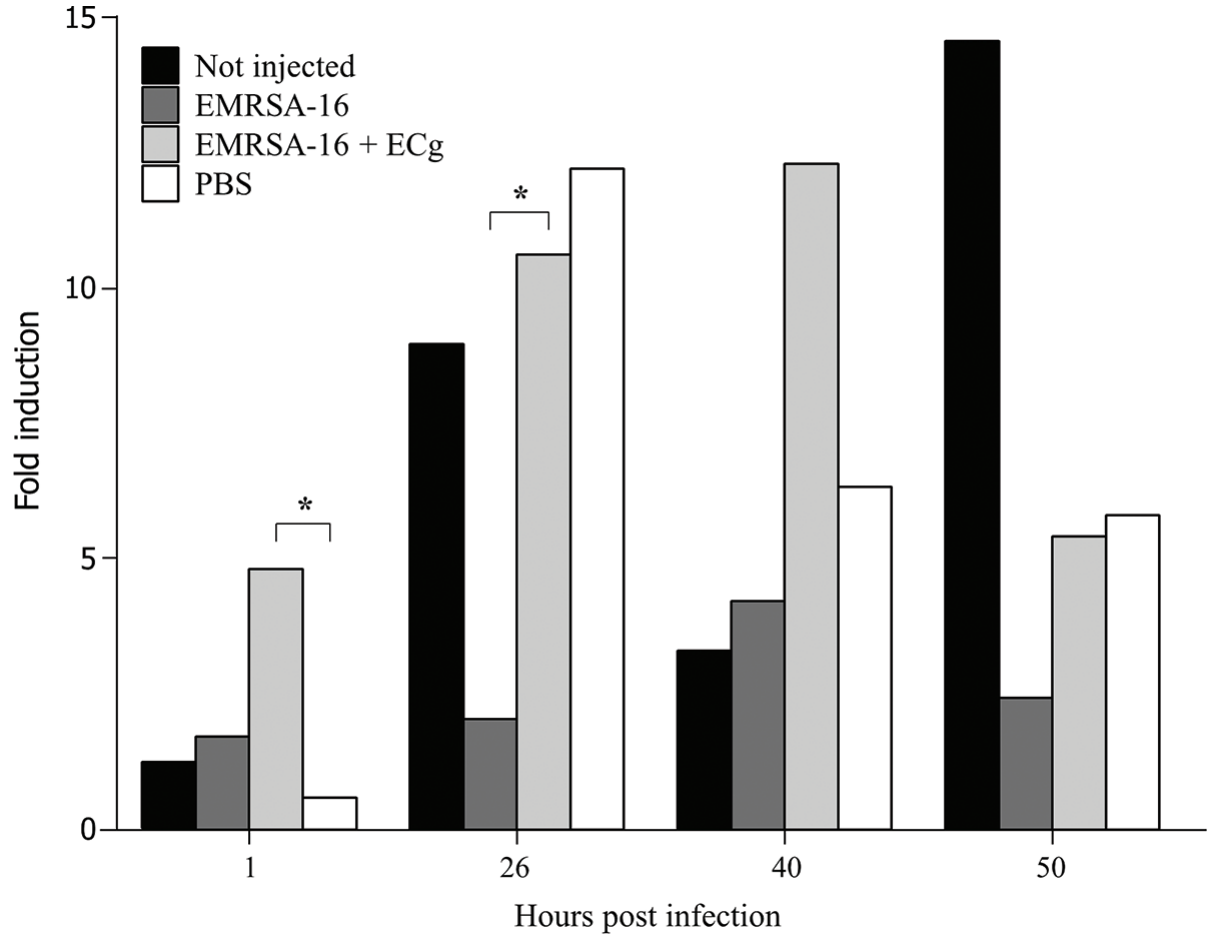
**Induction of the respiratory burst in 30 hpf zebrafish embryos following infection by the circulation valley with 1 × 10^4^ CFU EMRSA-16, 1 × 10^4^ CFU ECg-grown EMRSA-16 or PBS sham-injection control**. Embryos were induced with 400 ng/mL of phorbol myristate acetate in the presence of 1 μg/mL H2DCFDA and the intensity of the fluorescent signal measured as indicated. *n* = 10 per group; ^∗^*P* < 0.05; log rank test.

**FIGURE 6:**
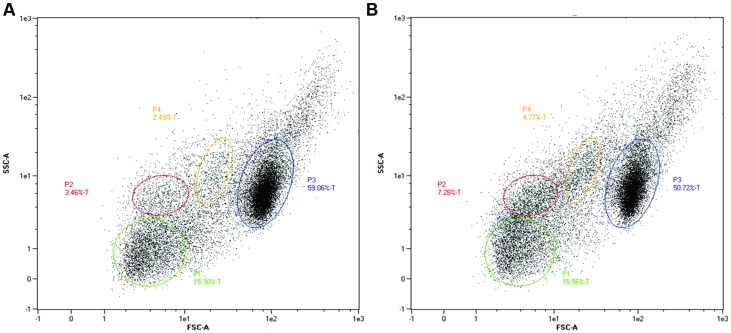
**Separation by flow cytometry (10,000 events) of subpopulations of blood cells at 17 hpi from zebrafish embryos infected by the circulation valley at 30 hpf**. Fluorescence reflects uptake by, or association with, GFP-labeled bacteria. **(A)** Embryos infected with EMRSA-16-GFP and **(B)**, with ECg-grown EMRSA-16-GFP. Gated populations P1-4 represent erythrocytes, lymphocytes, myelomonocytes, and immature precursors of all lineages. FSC, forward scatter (size); SSC, side scatter (changes to cell granularity). Blood cell populations identified by comparison with data from Hsu et al. (2004).

Uptake of injected EMRSA-16 by phagocytes suggests that the embryos mount a partially successful primary innate immune response to the challenge. We therefore determined the capacity of embryos to produce reactive oxygen species by measurement of the NADPH oxidase-dependent respiratory burst following injection of EMRSA-16 or ECg-grown EMRSA-16 into the circulation valley (**Figure 5**). At 1 hpi the response of embryos infected with ECg-grown EMRSA-16 was significantly higher compared to PBS-injected embryos; in contrast, there was no significant difference between controls and embryos infected with untreated EMRSA-16. At 26 hpi, embryos infected with untreated and ECg-grown bacteria displayed a significant response, although the respiratory burst associated with untreated EMRSA-16 was weaker than that found with embryos infected with ECg-exposed EMRSA-16. Sham-injection also induced a relatively large response but this was not significantly different from the untreated control. These associations were less pronounced at 40 hpi and were not evident at 50 hpi. These data suggest that ECg-grown bacteria were more readily countermanded by the innate defenses of the embryo, reflecting attenuated virulence following exposure to ECg. However, there were only small, insignificant changes in the distribution of the GFP label following uptake of EMRSA-16-GFP and the ECg-grown equivalent. **Figure 6** shows the distribution of label associated with 10,000 blood cells at 17 hpi of embryos infected at 30 hpf with ECg-grown and untreated bacteria.

There were no significant differences in the rate of transcription of the pro-inflammatory cytokine TNF-α as a result of infection of 30 hpf embryos with either EMRSA-16 or ECg-grown EMRSA-16 but levels of IL-1β gene expression were increased following infection (data not shown). At 2 hpi, IL-1β gene expression increased 2.7-fold and 1.6-fold in EMRSA-16-infected embryos and embryos infected with ECg-grown bacteria, compared to sham-treated controls. IL-1β gene expression in EMRSA-16-infected embryos further increased at 17 hpi, declining at 43 hpi.

## Discussion

Moderate concentrations of ECg substantially and reversibly alter the phenotype of MRSA isolates, compromising both virulence and β-lactam resistance without substantial repression of either the extent or rate of growth in batch culture (Stapleton et al., 2006). Translation of such *in vitro* data into efficacy in animal models of infection has potentially significant therapeutic value, through co-administration of resistance modifying agents with β-lactam antibiotics that have lost therapeutic utility due to the emergence of drug resistance. Further, the capacity of ECg and other catechin gallates to compromise virulence of MRSA suggests that they could be used as stand-alone anti-infective agents should such attenuation be evident *in vivo*. Therapeutic options would be enhanced if chemically stable, “druggable” analogs with increased potency and enhanced *in vivo* stability were available for profiling in mammalian models of infection but, as indicated in the Introduction current technical limitations to bulk synthesis of analogs of sufficient purity make this impracticable. Zebrafish embryos have been used as a model to investigate microbe-host interactions (reviewed by Goldsmith and Jobin, 2012) and fresh insight has been gained into the basis of host resistance to staphylococcal infection (Prajsnar et al., 2008). Further, zebrafish and their embryos are now widely considered as robust tools for drug discovery, drug development, toxicity profiling, and assessment of off-target drug effects (Chakraborty et al., 2009; Bowman and Zon, 2010; Strähle and Grabher, 2010). We therefore adapted the zebrafish embryo to investigate the capacity of ECg to modify the course of infection with the multi-drug-resistant epidemic clinical isolate EMRSA-16.

Zebrafish embryos have a number of attractions for initial low-cost *in vivo* profiling of therapeutic compounds: high fecundity and small size allow high throughput studies and the potential for the generation and storage of very large numbers of adults and embryos, optical clarity of the embryo permits visualization of structure in the living embryo, they have well-developed innate and adaptive immune systems with a high degree of conservation with mammalian systems and many drugs used to treat human diseases have comparable effects in embryos. There are, however, some disadvantages with respect to infection with human pathogens such as *S. aureus*: the optimum growth temperature range for staphylococci is 35–37°C whereas embryos must be infected and maintained at 28.5°C, it is difficult to dissect embryos for organ recovery for histology and for isolation of organ-specific RNA (Aleström et al., 2006), zebrafish do not possess mammalian organs such as skin, lung, mammary gland, and prostate (Kari et al., 2007) and their small size requires the injection of very small amounts of material which may lead to a high degree of variation between animals. In spite of these potential drawbacks, dechorionated embryos were readily infected with EMRSA-16 by injection into the circulation or yolk. Introduction of the pathogen into the circulation paralleled infection in mammalian hosts by this route, with a relatively large dose required to cause death. For example, Xiong et al. (2005) infected rats intravenously by tail vein injection with 10^4^, 10^5^, and 10^6^ CFU of *S. aureus* Xen29; animals dosed with 10^4^ CFU survived for 6 days, whereas one half infected with 10^5^ CFU died during this period. Mortality was observed in all rats infected with 10^6^ CFU within 6 days, showing that relatively large numbers of *S. aureus* are required to cause death when administered by this route. In contrast, relatively small numbers of bacteria (3–5 × 10^3^ CFU) exerted a lethal effect in embryos at or before 70 hpi when injected into the yolk sac at 30 hpf. This may be due to the physical nature of the yolk, which could hinder detection of bacteria by the host, whereas injection into the circulation allows for rapid immune detection of the pathogen.

We have investigated the capacity of ECg to increase the survival of embryos after EMRSA-16 infection. Green tea, rich in catechin gallates, is one of the most widely consumed beverages globally and its components are generally considered benign. However, there are reports that relatively high concentrations of ECg and other polyphenols exhibit some degree of toxicity when they are employed as a nearly pure compound free from interactions with other chemicals normally found in green tea extracts (Okabe et al., 1997; Babich et al., 2005); we therefore limited concentrations of ECg used in our study to 100 μg/mL to ensure that observed effects were unrelated to any inherent toxicity. Incubation of embryos in E3 medium containing quantities of ECg sufficient to attenuate EMRSA-16 and to convert bacteria to full β-lactam susceptibility had no impact on survival, even when oxacillin was incorporated into the incubation mixture at concentrations sufficient to kill ECg-modified bacteria *in vitro*. Further, a range of antibiotics at concentrations in excess of the MIC also failed to impact on embryo survival (Stevens and Taylor, unpublished data). The concentrations of ECg and oxacillin in the incubation mixture declined significantly during the course of these experiments, suggesting that these compounds may have absorbed to the surface of the dechorionated embryos but were unable to penetrate sufficiently to interact with the pathogen. Bolcome et al. (2008) indicated that selective inhibitors that work at nanomolar levels in cell-based assays should be used at 50- to 100-fold higher concentrations in zebrafish embryos to ensure penetration of the cell layers of the developing embryo. We therefore incrementally increased the concentration of ECg to 100 μg/mL, with again no impact on embryo survival. The permeability of zebrafish membranes has not been extensively investigated and the available evidence suggests that the rate of uptake of antibiotics and other compounds into embryos is highly variable. The chorion and yok sac are highly impermeable to aqueous solutes (Hagedorn et al., 1998; Kais et al., 2013), although chloramphenicol, but not kanamycin, penetrated the outer membrane to gain access to the internal matrix of dechorionated embryos (Song et al., 2010). Dechorionated embryos possess a thick outer coating which is likely to be a formidable barrier to penetration of a variety of molecules.

The amphipathic nature of catechins and catechin gallates imposes limits on their capacity to diffuse through biomembranes and other structural barriers. For example, they intercalate into the membrane of *S. aureus* and do not enter the cell (Bernal et al., 2010). It is therefore very plausible that their complete lack of efficacy when applied externally to embryos is related to their inability to cross numerous biological barriers to reach the staphylococcal target. In addition, the lack of any therapeutic effect from relatively high concentrations of antibiotics in the incubation mixture raises doubt over the value of embryos as tools for the screening of antibiotics, and potentially other therapeutic molecules. This issue needs further investigation as zebrafish embryos are finding increasing favor as an *in vivo* screen for new chemical entities.

This study has clearly shown that growth of EMRSA-16 in the presence of ECg produces a less virulent phenotype with a much lower capacity to elicit a lethal effect, avoid uptake and killing by phagocytes (as reflected in the respiratory burst) and generate a pro-inflammatory response by moderate induction of IL-1β. Thus, administration of ECg in a way that enables prolonged and effective contact with the target pathogen could lead to resolution of the infection. Parenteral administration of the polyphenol would shed light on this potential but an alternative to the zebrafish embryo model would be required. A similar conclusion was reached by Kao et al. (2010). They grew a drug-susceptible reference strain of *S. aureus* in the presence of grape seed extract, a natural food product rich in polyphenols, and injected the bacteria into anesthetized adult zebrafish; they noted significantly reduced inflammatory responses and mortality compared to bacteria grown in medium without supplementation. Thus, the zebrafish model has provided insights into the degree of attenuation attributable to ECg-mediated interactions in a robust *in vivo* model of infection but the capacity to effect this transition within the infected animal will require further studies in mammalian models of staphylococcal infection.

## Author Contributions

PT and CS conceived the experiments; PT, CS, HR, and RH designed the experiments; CS and RH performed the experiments; PT, CS, HR, and RH analyzed the data; PT wrote the paper.

## Conflict of Interest Statement

The authors declare that the research was conducted in the absence of any commercial or financial relationships that could be construed as a potential conflict of interest.
